# Epidermolysis Bullosa Acquisita: A Case Report of a Rare Clinical Phenotype and a Review of Literature

**DOI:** 10.7759/cureus.6386

**Published:** 2019-12-15

**Authors:** Cristina Beiu, Mara Mihai, Liliana Popa, Tiberiu Tebeica, Calin Giurcaneanu

**Affiliations:** 1 Oncologic Dermatology, Elias Emergency University Hospital, Carol Davila University of Medicine and Pharmacy, Bucharest, ROU; 2 Dermatopathology, Dr. Leventer Centre, Bucharest, ROU

**Keywords:** epidermolysis bullosa acquisita, blistering disorders, bullous diseases

## Abstract

Epidermolysis bullosa acquisita (EBA) is an autoimmune subepidermal bullous disorder of the skin and mucous membranes. The disease results from the production of immunoglobulin G (IgG) antibodies against type-VII collagen, a major component of anchoring filaments in the dermal-epithelial junction. The disease has two major forms of presentation: the classical (non-inflammatory) type and the inflammatory type. Classical EBA is mainly characterized by the following features: development of non-inflammatory tense blisters on trauma-prone areas, multiple milia cysts, minimal or no inflammation findings on histopathology. Alternatively, inflammatory EBA is defined by widespread inflammatory blistering eruptions and a neutrophil-rich inflammatory infiltrate on standard histopathology. In both cases, specialized immunopathological findings are further required to establish an accurate diagnosis. In this article, we present an atypical case that shares features of both inflammatory and non-inflammatory forms of EBA. The case also serves to review and synthesize current concepts on the etiopathogenesis, diagnosis, and treatment of this extremely rare disease.

## Introduction

Epidermolysis bullosa acquisita (EBA) is an acquired, subepidermal mucocutaneous blistering disorder that results from autoimmunity to collagen VII, a main structural component of anchoring fibrils in the basement membrane zone (BMZ) of the dermal-epidermal junction (DEJ). The incidence of this rare disease is approximated at 0.2 per one million people and it usually affects middle-aged adults [1]. Anecdotally, the disease exhibits two main clinical and histopathological forms: non-inflammatory (also called "classical form") and inflammatory EBA, the latter mimicking other subepithelial autoimmune blistering disorders [2]. 

This case illustrates an atypical clinical phenotype of EBA presenting with specific clinical findings of the classical form but with unequivocal histopathological features of inflammatory EBA. The case also serves to review classic and unexpected findings of the etiopathogenesis, diagnosis, and treatment of this extremely rare disease.

## Case presentation

**Figure 1:**
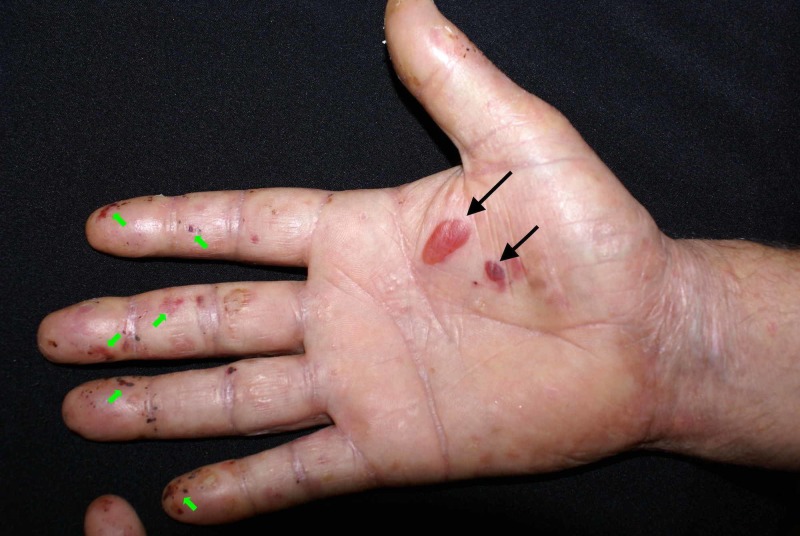
Clinical image illustrating tense blisters (black arrows) and multiple erosions (green arrows) on the right palm

**Figure 2:**
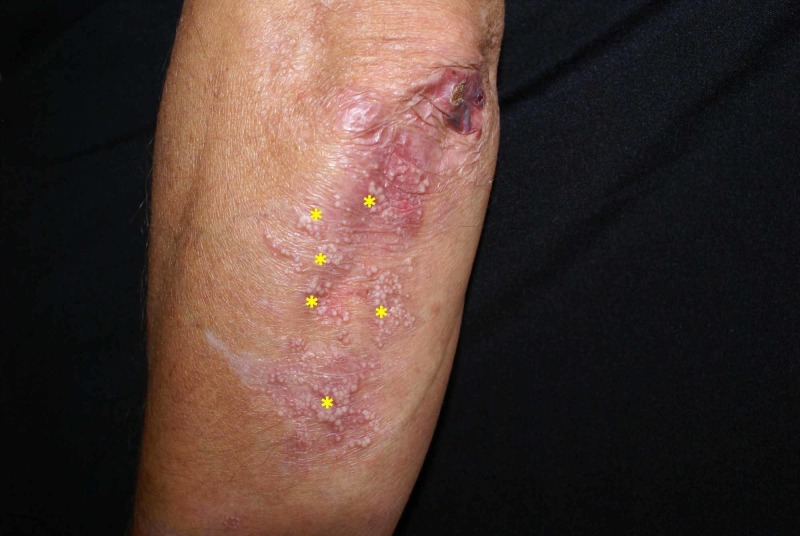
Multiple milia cysts (yellow asterisk) developed on an older lesion on the elbow

**Figure 3:**
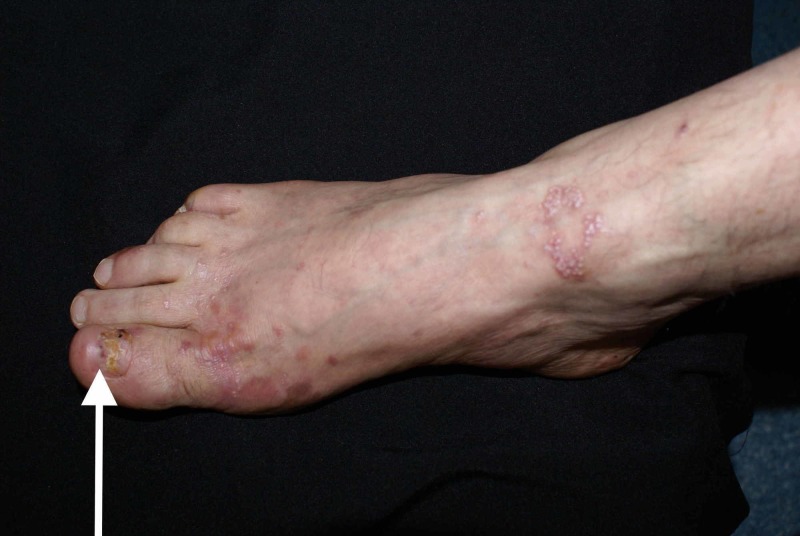
Marked onychodystrophy of the big right toenail (white arrow)

**Figure 4:**
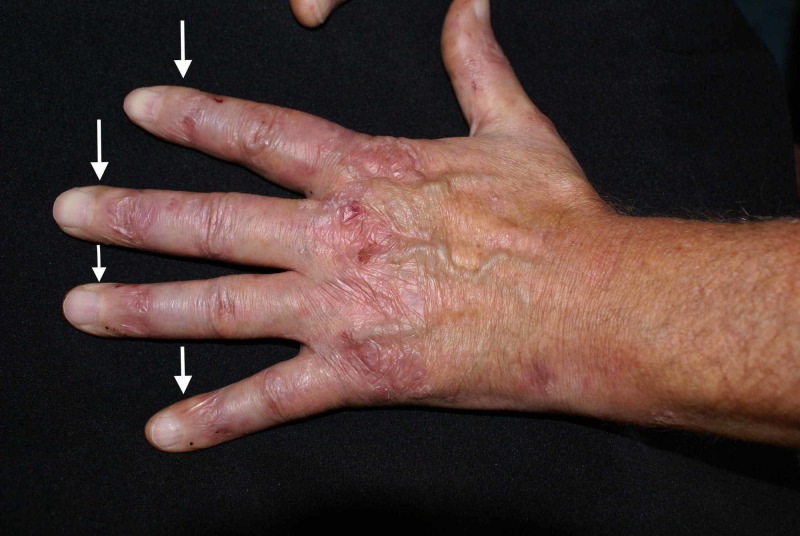
Fibrotic changes of the fingers; please notice the shiny and thickened aspect of the skin (white arrows)

**Figure 5:**
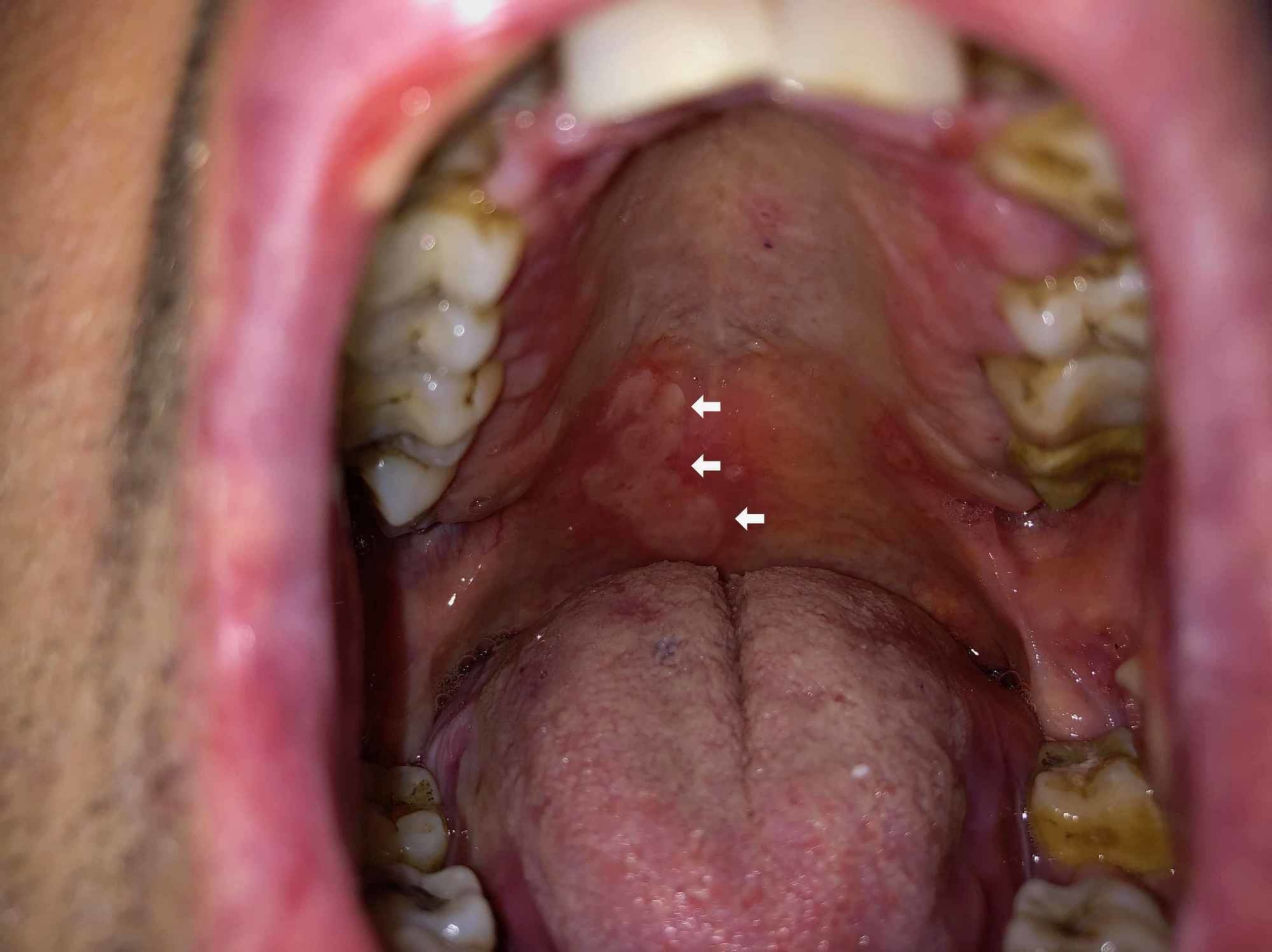
Mucosal erosions on the palate (white arrows)

A 54-year-old Caucasian male presented to our dermatology department for the evaluation of a mucocutaneous blistering eruption that had evolved over a period of three years. The eruption consisted of tense blisters that easily rupture to form painful erosions (Figure 1). Some of the older erosions had already healed with small atrophic scar areas and multiple milia cysts (Figure 2). The patient had complaints of increased skin fragility stating that the lesions were easily induced by minor injuries. The lesions were widespread but indeed had a predilection for areas that are regularly prone to repetitive trauma: palmoplantar area, elbows, knees, and posterior trunk. Physical examination additionally showed onychodystrophy with partial loss of the big right toenail (as seen in Figure 3) and moderate fibrosis of the fingers, with reduced hand mobility (Figure 4). The patient also suffered from concomitant mucosal involvement, with multiple oral erosions (Figure 5).

Prior to referral in our clinic, the patient was initially diagnosed as having bullous pemphigoid (BP). A review of the patients' previous medical records showed that the diagnosis was based on direct immunofluorescence studies of a biopsy section which revealed the deposition of immunoglobulin G (IgG) and C3 at the DEJ in a linear pattern. 

We performed a comprehensive metabolic panel which was within normal limits. Pemphigoid circulating antibodies (BPAG 180 and BPAG230) and antinuclear antibodies (ANA) were all negative and C3 and C4 were within the normal range. An esophago-gastro-duodenoscopy showed extensive erosions on pharyngeal and upper-esophagus mucosa. No stricture or stenosis was detected. A colonoscopy was also performed but no signs of inflammatory bowel disease were detected. A thorough review of systems was entirely negative. 

**Figure 6:**
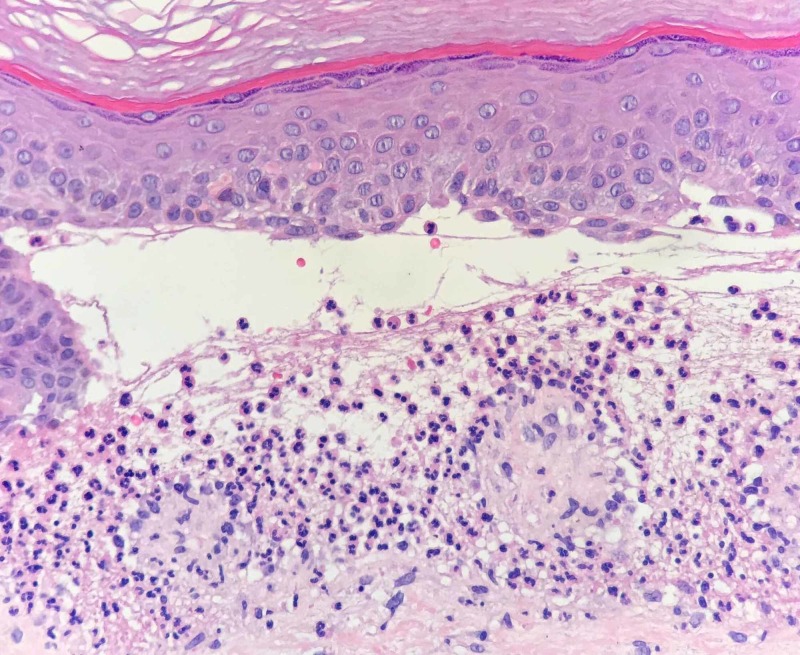
Microscopy image (Hematoxylin and Eosin staining) showing subepidermal blistering and a neutrophil-rich infiltrate in the papillary dermis and within the bullous lesion

Two 4-mm punch biopsies were taken, one lesional for hematoxylin and eosin (H&E) and one perilesional for direct immunofluorescence (DIF). Standard histopathology with H&E showed subepidermal blistering with a neutrophil-rich infiltrate in the papillary dermis and within the bullous lesions. Mononuclear cells such as lymphocytes and monocytes could also be observed. Discrete fibrous changes of vascular hyperplasia were present in the superficial dermis, representing the histopathological correlation of the clinical scarring (Figure 6). 

**Figure 7:**
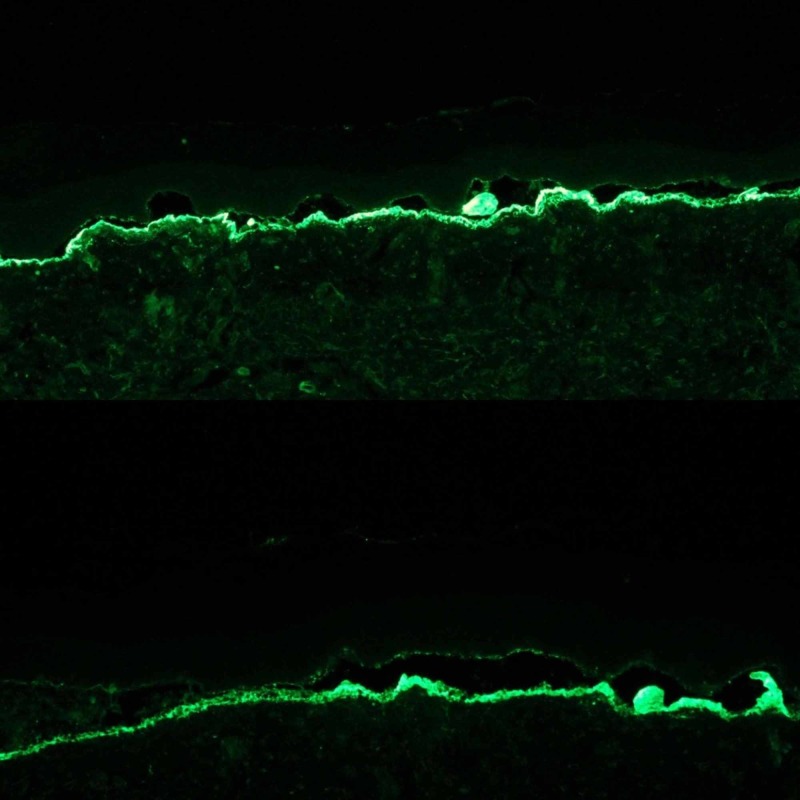
Studies on “salt-split skin” demonstrated linear deposits of the immunoreactants, mainly IgG, on the dermal side of the dermo-epidermal separation, at the base of the bullae

Direct immunofluorescence tests showed linear deposits of IgG and C3 at the DEJ. IgA tested negative. Fibrinogen was positive in the cleavage area (non-specific finding). The "salt-split skin" technique showed the localization of the immunoreactants, mainly IgG, along the dermal side of the artificially induced blisters at the level of lamina lucida (Figure 7). 

These findings supported the clinical diagnosis of EBA. We initiated treatment with 50 mg of dapsone per day. This resulted in decreased formation of new blisters. However, disease activity has persisted. 

## Discussion

The two most common forms of EBA are: (1) the classical (non-inflammatory) type and (2) the inflammatory type, subsequently divided into various subtypes: (i) BP-like EBA; (ii) clinically mimicking cicatricial pemphigoid-like; (iii) linear IgA bullous dermatosis (LABD)-like; (iv) and Brunsting-Perry pemphigoid-like. The two most common presentations are the classical non-inflammatory EBA and the BP-like EBA [2].

The classical form is characterized by tense blisters and erosions without clinically associated inflammation. It is usually localized towards trauma-prone areas. Millia and scarring are common findings. In severe cases, nail loss, fibrosis of the fingers and esophageal stenosis may occur. In contrast, the inflammatory form is characterized by vesiculobullous disseminated eruptions with a predilection for the trunk, intertriginous areas, and extremities. The tense bullae develop on erythematosus, inflamed basis [3]. Mucosal lesions are frequent in both classical and inflammatory EBA and both ocular and gastrointestinal mucosa can be affected in various degrees [4]. 

From the clinical features, we initially considered our patient as having classical non-inflammatory type EBA. But histopathology showed numerous neutrophils within the subepidermal blisters as well as in the interstitial infiltrate, pointing towards an inflammation-rich form [5]. Considering both the clinical and histopathological aspects, we hypothesized that our patient suffered from an atypical form of EBA, a mixed form that seems to have resulted from an overlap between the classical and the inflammatory forms. A possible explanation for this mixed clinical phenotype would be the epitope spreading phenomenon that has been extensively investigated in autoimmune skin diseases. Numerous epitopes on the N-terminal domain of type VII collagen are identified by circulating autoantibodies in patients with EBA and reactivity with various epitopes can induce different clinical phenotypes [2,6]. 

While clinical and standard histopathological findings can be characteristic for EBA, they certainly are not specific and additional testing is required. The diagnosis can be narrowed down with the aid of direct immunofluorescence (DIF) testing for tissue-bound autoantibodies. This involves immunofluorescence labeling of antibodies. On DIF, we basically tag the patient’s autoantibodies on tissue sections and this would show linear deposition of IgG and often C3 along the BMZ. But this is still a non-specific finding, as it can be seen in BP as well as a number of other subepidermal autoimmune blistering disorders [7]. 

So the best next test to perform would be immunofluorescence on BMZ-split skin. Perilesional tissue is incubated with 1 mol sodium chloride (1 M NaCl) and induces an artificial split (cleavage) at the level of lamina lucida, a particularly vulnerable area of the basement membrane. Subsequent binding of autoantibodies on the dermal or epidermal side of the induced bullae helps refine and narrow the differential diagnosis [8]. When we consider the anatomy of the basement membrane, collagen VII is located at the bottom, as part of the anchoring fibrils, underneath lamina densa and lamina lucida. So, we expect that the target of the autoantibodies involved in EBA would be below the lamina lucida, resulting in a linear deposition of IgG and potentially C3 at the base of the artificially induced salt-split skin (on the dermal side). This can also be seen in some forms of mucous membrane pemphigoid, as well as bullous systemic lupus erythematosus (SLE) so additional tests may be required if the later disorders are suspected [9-10]. In contrast, in BP, which is often the primary differential diagnosis for EBA, linear deposits of IgG are seen on the roof of the induced blister (on the epidermal side) or on both the dermal and epidermal side simultaneously [10]. 

The "salt-split skin" technique can be performed by utilizing perilesional skin (DIF), as in our patient’s case, or by using serum from the patient and a salt-split human skin as a substrate (indirect immunofluorescence). A drawback for the use of IIF is represented by the fact that some patients may have low serum levels of type VII collagen antibodies, resulting in false-negative test results [11]. 

In our case, and most of the cases in general, the diagnoses of EBA can be established through the correlation between the clinical findings, pathological features and immunoreactivity pattern in the "salt-split skin" technique [3]. On a research basis, additional testing is available to further differentiate EBA from other rare sub-lamina-densa blistering diseases. Examples of such tests are: (I) Transmission electron microscopy -shows the blister cleavage below the lamina densa and also a decrease in anchoring fibrils at that level [12]; (II) Enzyme-linked immunosorbent assay (ELISA) - a quantitative test that can detect and also monitor the level of collagen VII autoantibodies specifically [13-14]; (III) Direct and/or Indirect Immunoelectron microscopy (IEM) - these tests can provide highly precise ultrastructural location of IgG deposits in the perilesional skin. The IgG antibodies are previously labeled with colloidal gold or peroxidase and then appear as electron-dense deposits on the anchoring fibrils [15]. 

As shown in our case, EBA itself can cause considerable morbidity. But it can also be associated with a variety of systemic conditions such as Crohn’s disease, SLE, or several endocrinopathies [16]. Inflammatory bowel disease (IBD) is the most common associated disease. A possible explanation is that type VII collagen is also expressed on the basement membrane of the human colon and autoantibodies against type VII collagen were found in some patients with IBD [17]. Therefore, it’s important to have a high level of suspicion if patients have a positive review of systems beyond the skin.

When it comes to treatment options for EBA, data are limited. Because it is a very rare condition, there is a lack of well-designed or large studies such as randomized control trials investigating treatment options for this condition. So we’re left with a handful of case reports to help guide treatment decisions. Often empiric treatment is tried with patients and different medications are introduced in a stepwise approach depending on the patient’s response. Unfortunately, the disease tends to be fairly refractory to treatment but the inflammatory forms do tend to do a little bit better [16].

Long-term systemic glucocorticoids have proven to be less effective for EBA than for other blistering disorders. Some improvements have been documented with immunosuppressive agents [18]. For the previous three years, our patient had received treatment with oral glucocorticoids and systemic immunosuppressive medications, including azathioprine, cyclosporine or methotrexate, with no significant improvement. Thus, we preferred a different approach to initial therapy. We initiated treatment with dapsone, since treatment recommendations for most patients consist of colchicine or dapsone, in monotherapy or in combination [18-19]. For patients with EBA refractory to colchicine and dapsone, rituximab, an anti-CD20 monoclonal antibody, is a promising option but the high costs represent a major barrier in the use of these agents for patients with EBA [14]. 

A curative treatment for EBA does not currently exist. Hence, the goal of treatment is long-term remission of the disease. It usually has a prolonged course [18]. 

## Conclusions

We conclude that in the case of EBA, and in other rare diseases in general, current knowledge could be enriched with the discovery of additional clinical phenotypes that at the moment may be under-diagnosed or under-reported in the literature. Further studies will be required to clarify whether our findings are simply hypothetical or purely incidental, or if EBA is indeed a disease with further various subtypes to be unraveled and well-established. 
